# Making connections: teaching and learning bioelectrochemistry

**DOI:** 10.1007/s00775-025-02131-y

**Published:** 2025-12-24

**Authors:** James P. McEvoy

**Affiliations:** Department of Biological Sciences, Royal Holloway University of London, Egham, Surrey, TW20 0EX UK

**Keywords:** Electrochemistry education, Bioelectrochemistry, Redox biology, Pedagogy, Curriculum, Teaching and learning

## Abstract

Advances in bioelectronic and renewable energy technologies make it important for undergraduate students to learn about bioelectrochemistry, and courses in the chemical and biological sciences provide opportunities for them to do so. This review surveys the ways in which bioelectrochemistry appears in these degree programs and evaluates effective educational practices in each case. Three key pedagogical challenges are identified: bioelectrochemical concepts are often abstract, the terminology is confusing and variable, and relevant material is fragmented across the curriculum. The review makes five recommendations to address these challenges. Educators are advised to (1) signpost connections across the curriculum to promote topic integration, (2) provide explicit guidance on terminology to pre-empt confusion, (3) use vivid biological examples to capture student attention and interest, (4) use active learning techniques to strengthen conceptual links, and (5) adopt published laboratory exercises that allow students to put bioelectrochemical theory into practice. In each case, examples are given to support implementation and enhance undergraduate student appreciation of the field.

**Graphical abstract:**

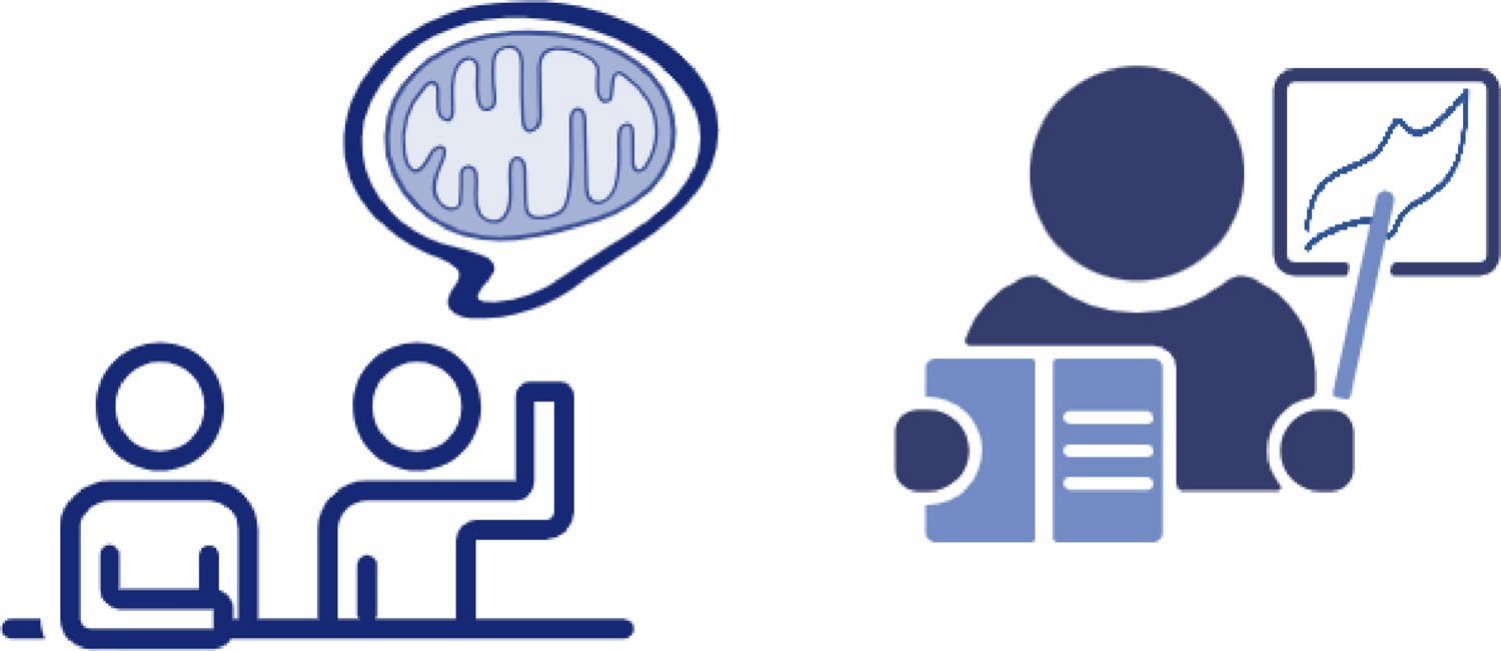

## Introduction

Recent years have seen increasing interest in undergraduate electrochemical education [1, 2], with several articles published on the subject [3–5]. These works emphasize the need to train students for areas of contemporary importance such as energy conversion and storage, electrocatalysis, and electroactive materials science. Others highlight the need to develop the emerging fields of synthetic organic electrochemistry and electrochemical drug discovery [6].

Electrochemical educators at the undergraduate level face three challenges [3]. First, the content is inherently difficult. Even the simplest concept, the reduction potential, is abstract for many students, and mastery requires the quantitative application of many such ideas. Second, electrochemical language and notation can be obscure, and includes antiquated terminology such as “electromotive force.” Third, electrochemical and redox topics are scattered throughout the curriculum, appearing in different guises even within the same degree course. This fragmentation makes it hard for students to reach an integrated understanding. Misconceptions from high school often persist, compounding the problem. For instance, students often believe that electric currents are carried through solutions by electrons, or that the anode in an electrochemical cell is always positively charged [7, 8], and such errors may be reinforced by their university textbooks [9].

Everything written about the importance of electrochemical education applies even more to biological electrochemistry. Its technological and economic significance makes it a crucial part of contemporary scientific education: bioelectrochemical systems underpin biosensors, biofuel cells, redox microbiology, biomining, bioremediation and bioelectronic interfaces, including wearable and implantable devices [10]. As for the discipline’s difficulty, it asks students to do everything that electrochemistry demands − apply electrochemical concepts, interpret electrochemical jargon and integrate material from different parts of the curriculum – as well as to understand the relevant biology.

Despite its importance and its challenges, biological electrochemistry has been neglected in the educational literature. For example, in a recent special collection entitled *Electrochemistry & Education*, whose scope included biochemistry, just 4 out of 56 papers addressed biological applications [2]. The reason for this is clear. Electrochemistry, for all the obstacles it presents to educators, is recognized as a distinctive area of chemistry, and chemistry students (as well as some other physical scientists) are expected to learn it. Bioelectrochemistry, on the other hand, is an interdisciplinary field whose practitioners work in different departments, and it is explicitly considered only at the postgraduate level.

Undergraduate bioelectrochemistry education may go unrecognized, but it does happen. Employing an appropriately broad definition of the field – the use of electrochemical principles and methods in a biological context – we find that bioelectrochemistry is widely encountered by undergraduate students in both the physical and the biological sciences. This matters for two reasons. First, far more students encounter bioelectrochemistry as undergraduates than as postgraduates, and they carry their impressions of the discipline into their careers. Bioelectrochemical instruction in bioscience courses has a particularly wide reach. In the UK, for instance, nearly 90% more undergraduates study biological sciences than physical sciences [11]. Second, it matters to bioelectrochemical research and the talent pipeline: improvements in how undergraduates learn bioelectrochemistry will mean that more of them will be inspired to continue their studies and contribute to the discipline.

This review examines the ways in which bioelectrochemistry is taught in undergraduate chemistry and biological science courses, identifies some challenges and good pedagogical practice, and offers five recommendations for teachers. Although most curricula considered are from the UK and the USA, published practice spans various university systems, making the advice broadly applicable.

### Bioelectrochemical education in the chemical sciences

Electrochemistry forms part of the core curriculum in almost all UK chemistry degrees, and is taught in both general and physical chemistry modules [3]. In the USA, electrochemistry is prominent in analytical chemistry classes [12]. Advanced inorganic chemistry modules, through the study of bioinorganic chemistry and protein electron transfer [13], give students more exposure to bioelectrochemistry at some institutions.

Introductory bioelectrochemical content is suggested by popular general chemistry textbooks, which often mention the biological applications of electrochemistry in case-study boxes or historical asides. For example, in the electrochemistry chapter of one widely used general chemistry textbook, a box entitled *Heartbeats and Electrocardiography* explains cardiac potentials and ECGs; elsewhere there is a photograph of an electric eel to add zoological interest [14]. Of more molecular significance is a textbook’s box on respiratory electron transfer, which includes a picture of an iron-sulfur cluster [15]. Another general chemistry textbook, in an outstanding example of biological salience, begins its electrochemistry chapter with an overview of photosynthesis and describes attempts to make an artificial leaf [16].

The history of electrochemistry provides further biological examples. Often mentioned are Galvani’s electrical experiments on frogs’ legs and Von Humboldt’s experiments on electric eels [17], which inspired the early electrochemical work of Volta, Davy, Faraday and Daniell [14, 18]. This early connection between electrical and biological research emphasizes the link between what we now know as electrochemistry and electrophysiology [19]. Judicious use of scientific history can increase students’ knowledge of a subject and help them appreciate the way in which scientific knowledge is gained [20], and this approach may be valuable in electrochemical education [21].

Besides historical narratives, many other connections between electrochemistry and biology can engage introductory students and add educational value in the affective domain [22]. Contemporary bioelectrochemical “hooks” may be found in the work of well-known companies [23, 24] and research into the origins of life [25]. Practical demonstrations can also play their part: for example, students may be shown how redox chemistry links respiring yeast and a hydrogen fuel cell [26, 27]. The lemon cell is the best known demonstration of the connection between electricity and biology. It is a mainstay of school science outreach [28] and has even been used at the undergraduate level [29], but it should be handled with care. Children may think that the lemon itself generates the electrical potential, the same erroneous conclusion that Galvani drew from his frogs’ legs [19]. Its use of zinc and copper electrodes can lead older students to believe that the lemon cell works like the Daniell cell, which it does not [30].

Bioelectrochemical practical exercises appear to be rare in the undergraduate electrochemistry laboratory, appearing in just 3 out of 18 recently surveyed examples [3]. This impression is reinforced by the electrochemical experiments that have been published in chemical education journals. These generally focus on inorganic [31–33] or organic [34, 35] chemistry, while the physical chemistry aspects of the topic are often taught with computer simulations [36–39].

**Fig. 1:**
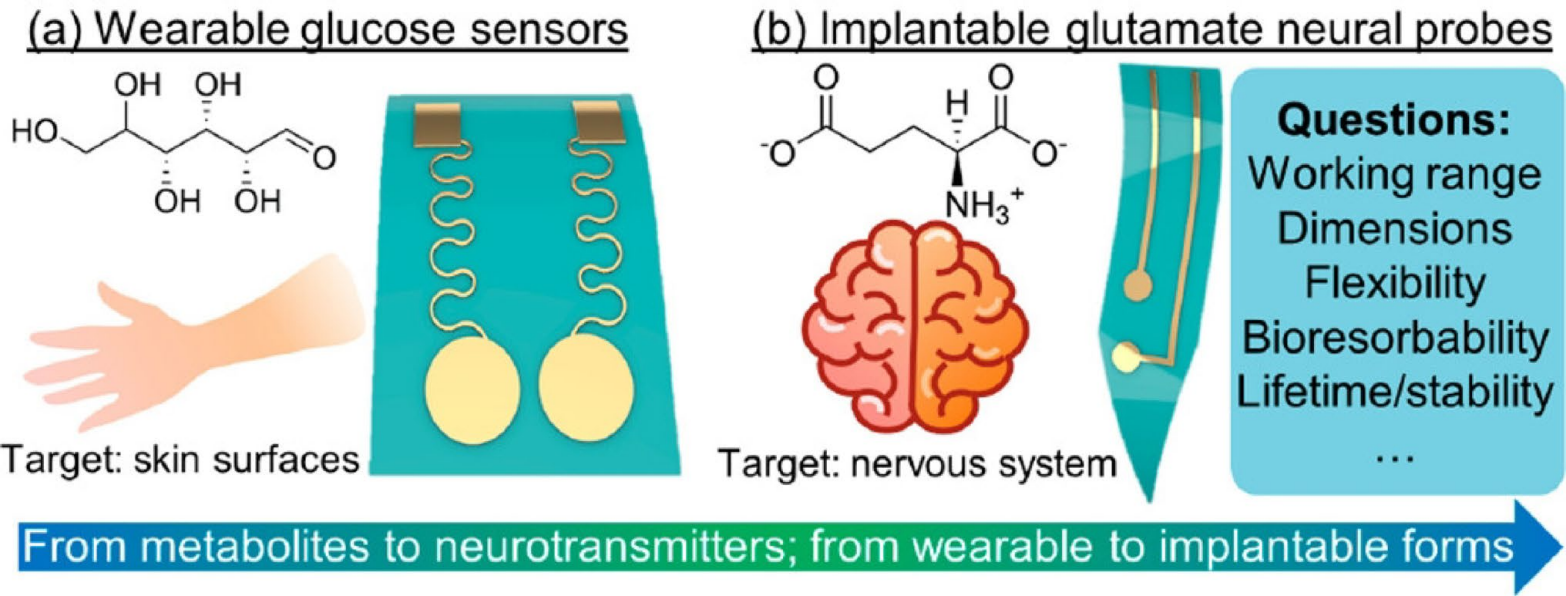
Conceptual framework of a multidisciplinary teaching laboratory in which students (**a**) prepare stretchable electrodes using a cut-and-paste method, functionalize their surfaces for glucose voltammetry, and characterize wearable sensor performance; (**b**) explore the design of glutamate neural probes. Reproduced with permission from [43]

The few published practicals that deal with the electrochemistry of biological molecules in the advanced undergraduate chemistry laboratory fall into two categories. Experiments in the first set are bioanalytical. In these exercises, which link to biosensor development and other health applications [40], students use dynamic electrochemical techniques to study and quantify small biological molecules such as glucose, dopamine, ascorbic acid, tyrosine and acetaminophen [41–43]. Figure 1 illustrates one such laboratory: students prepared and characterized biointegrated glucose sensors over three practical classes, extending to glutamate detection in the fourth [43]. Dopamine and glutamate are important analytes because their roles in neurophysiology can be studied by in vivo voltammetry [44, 45].

The second, smaller set of published bioelectrochemistry experiments deals with the redox properties of proteins, particularly cytochrome *c* [46, 47]. Students at universities with bioelectrochemical research strengths sometimes carry out protein film voltammetry in an advanced laboratory class [3], and the results of undergraduate research projects using the same technique have been published [48]. To support the introduction of protein electrochemistry into their own teaching laboratories, educators may find useful a popular undergraduate tutorial on cyclic voltammetry [49], a primer on protein film voltammetry [50], and a case study on integrating practical electrochemistry into undergraduate curricula [51].

### Bioelectrochemical education in the biological sciences

Since electrochemical content in undergraduate biological science curricula has not been surveyed before, the requirements of some UK Professional, Statutory and Regulatory Bodies (PSRBs) were consulted. The Quality Assurance Agency for Higher Education (QAA) notes in its Biosciences subject benchmark statement that some bioscience courses will overlap with the Chemistry benchmark [52]. However, neither the QAA Chemistry benchmark statement [53] nor the Royal Society of Chemistry’s degree accreditation handbook [54] mentions electrochemistry at all. The Royal Society of Biology (RSB), in contrast, is quite detailed in its subject-specific accreditation requirements for degrees using ‘Biochemistry’ in their title, asking that these courses should include a “sensible grounding” in “thermodynamics, particularly of solutions, including electrochemistry” [55]. The RSB accredits hundreds of UK bioscience degrees [56], and those with a molecular emphasis will often share introductory modules with the department’s Biochemistry programme. The counterintuitive consequence is that a British university may face more pressure from PSRBs to teach electrochemistry to its bioscience students than to its chemistry students.

**Fig. 2:**
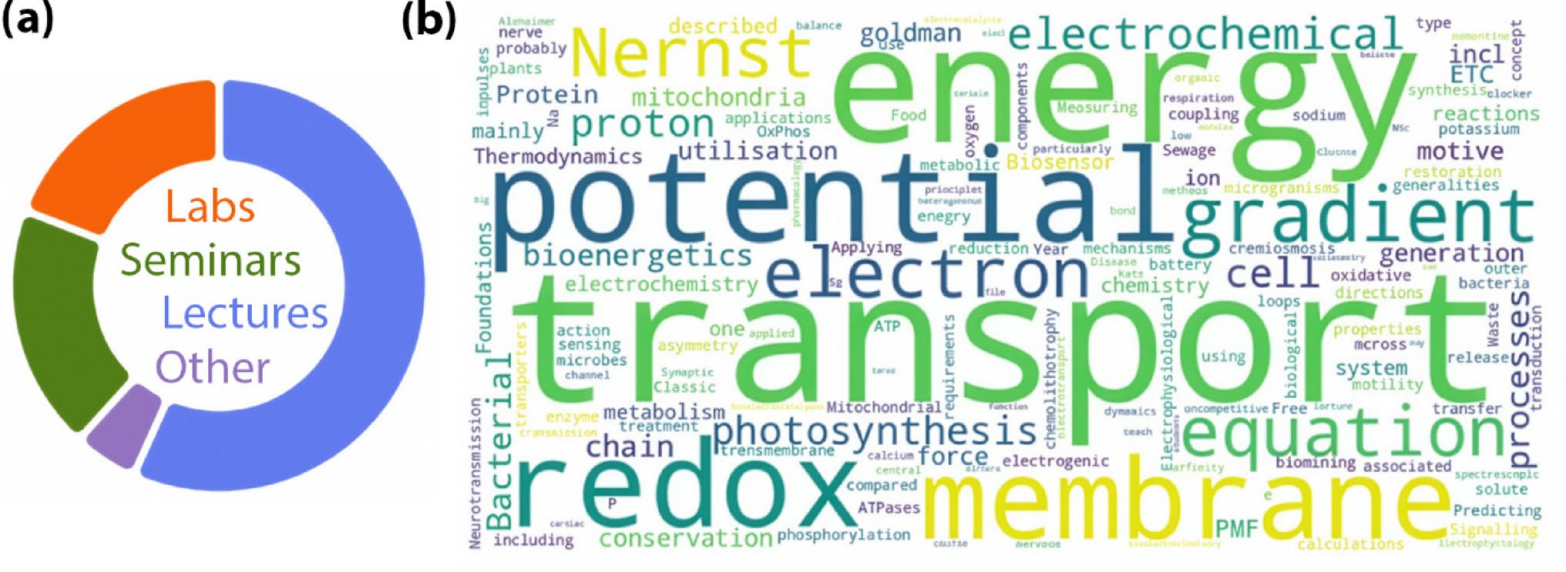
(**a**) Reported teaching methods in a small informal survey of bioelectrochemical topics (Lectures *n* = 12; Laboratory Practicals *n* = 4; Seminars/Workshops *n* = 4; Other *n* = 1, described as a software simulation). (**b**) Word cloud of free-text responses to: “Briefly describe the bioelectrochemical topics that you think are covered in your bioscience degrees.”

The prevalence of bioelectrochemistry topics in biological science curricula is illustrated by a small, informal survey conducted by the author. Colleagues at 12 universities (9 in England, 2 in the USA and 1 in the Netherlands) reported that electrochemical principles and methods were taught across a wide range of their degree courses, including Biochemistry, Biology, Biomedical Sciences, Biomedicine, Cell Biology, Life Science and Technology, Medical Biochemistry, Neuroscience, Pharmacology, and Pharmacy. Bioelectrochemical content appeared at each degree stage and in various modules, including Biochemistry, Biophysical Chemistry, Chemistry (General, Inorganic and Physical), Cell Biology, Cell Membranes and Bioenergetics, Cellular and Molecular Neuroscience, Drug Development, Environmental Microbiology, Microbial Biotechnology, Neuronal and Cellular Signalling, Mammalian Physiology, and Nutrition and Metabolism. Lectures were the most frequently cited teaching method, while seminars/workshops, practical laboratory exercises and computer simulations were mentioned less often. Figure 2 shows the proportions of teaching methods reported and a visual summary of bioelectrochemical topics mentioned by respondents.

Introductory chemistry classes for bioscience students present the fundamentals of redox chemistry in the context of biological thermodynamics and equilibria [57]. Case studies may include the glucose oxidase-based electrochemical glucose sensor and the physiological effects of reactive oxygen species [57, 58]. It is hard to run practical exercises in these areas because introductory bioscience classes often contain hundreds of students and the equipment that might be used is expensive; first-year bioscience students are therefore more likely to quantify glucose spectrophotometrically than electrochemically [59]. However, low-cost electrodes [60] and potentiostats [61–64], along with open-source software for data acquisition and analysis [65, 66], make bioelectrochemistry practicals increasingly feasible for large cohorts.

Bioinorganic chemistry, which is also required for Biochemistry degree accreditation by the RSB [55], provides another route to bioelectrochemistry education. Sodium and potassium’s role in establishing membrane potentials, as well as iron’s role in oxygen transport, are both typically included in this topic [57, 58]. In the laboratory, students can use conductivity meters to quantify the solution behaviour of biologically-relevant electrolytes [67, 68] and use visible spectrophotometry to characterize different redox states of hemoglobin [69]. With appropriate equipment this experiment may be extended to hemoglobin’s voltammetric analysis [70].

Undergraduate bioscience students learn more about membrane potentials in their introductory physiology classes, and the topic is revisited at a more advanced level in cell biology, pharmacology and neuroscience modules. The theory of electrophysiology rests upon the Nernst equation, which can be used to calculate a cell’s resting membrane potential [71, 72]. This equation should provide a useful link between the electrophysiological and electrochemical parts of the bioscience curriculum, and some general chemistry texts do make this connection [18]. Its usefulness is diminished, though, by important differences in the way the equation is used by chemists and biologists. These differences threaten to confuse students if they are not properly appreciated.

In their introductory chemistry classes, bioscience students are taught to use the Nernst equation to calculate the cell potential *E*_cell_ under thermodynamically non-standard conditions, and it is typically written as Eq. 1 [18]:

1 $$\:{E}_{cell}={{E}_{cell}}^{^\circ\:}-\frac{RT}{nF}\mathrm{ln}Q$$

where *n* is the dimensionless number of moles of electrons transferred in the cell reaction, *Q* is the reaction quotient (approximated in general chemistry classes as a ratio of concentrations) and the other symbols have their usual meanings. In their cell biology and physiology classes, on the other hand, students are taught to use Eq. 2, a different version of the Nernst equation, to describe the equilibrium resting potential of a single ionic species [73]:

2 $$\:{E}_{ion}=\frac{RT}{ZF}ln\frac{{\left[ion\right]}_{out}}{{\left[ion\right]}_{in}}$$

where *Z* is the charge on the ion of interest. According to Eq. 2, the equilibrium membrane potential is that at which the ion experiences no electrochemical driving force across a membrane permeable only to this species. Applying this version of the Nernst equation to potassium ion concentrations approximates the resting potential of an animal cell’s plasma membrane, because its potassium channels are open and its sodium, chloride and calcium channels are mostly closed. Advanced students use the Goldman equation, an extension of the Nernst equation which includes other ions and their respective permeabilities, to calculate resting membrane potentials [74].

Those who teach bioelectrochemistry to bioscientists should expect three difficulties to surround the Nernst equation in their students’ minds. The first is the practical, mathematical challenge of using the equation, particularly the logarithmic term [5, 75]. This is compounded by asking students to use different versions of the equation without explanation. The second difficulty is conceptual, and relates particularly to their understanding of what is meant by an electrochemical potential [76, 77]. The third difficulty is terminological, and comes from the different ways in which chemists and biologists understand the term “equilibrium potential”.

Chemists hardly ever describe the reversible, open-circuit potential difference of an electrochemical cell as an “equilibrium potential”, preferring “cell potential” [18] or, in some contexts, “open-circuit voltage” [12]. They take particular care to avoid the word “equilibrium” when they are teaching electrochemistry in the context of chemical thermodynamics, because a galvanic cell is not at thermodynamic equilibrium. (Local electrochemical equilibrium is established at each electrode, but that is not the same [78].) The potential difference of an electrochemical cell that has reached thermodynamic equilibrium is trivially zero: students are taught in their introductory chemistry classes that “a ‘dead’ battery is one in which the cell has reached equilibrium” [18]. Electrophysiologists, on the other hand, *do* describe membrane potentials as “equilibrium potentials”, meaning a special case of electrochemical equilibrium: the balancing of the free energy term due to a species’ concentration gradient against an opposing membrane potential energy term [72, 73]. This membrane potential depends on actively-maintained concentration gradients, maintained in particular by the sodium-potassium pump, and the overall system is therefore far from thermodynamic equilibrium. Students are likely to find the term “equilibrium potential” confusing unless they have been warned about this context-dependent difference in terminology.

There are various practical exercises to help bioscience students develop an integrated understanding of membrane potentials. A galvanic concentration cell has been found useful at the high-school level [79], and may prove so in a university chemistry module if its physiological significance is emphasized. More realistic in vitro cell models using semi-permeable membranes have been used effectively [74, 80]. In pharmacology modules, the in vivo effects of action potentials under the influence of various drugs can be measured in the ilea of small mammals [81, 82]. Advanced students of electrophysiology can carry out fast scan cyclic voltammetric analysis of neurotransmitters [66] and benefit from sophisticated computer simulations [83].

Biochemistry and bioenergetics modules are fertile ground for bioelectrochemical education, particularly in the context of energy metabolism. Electrochemical concepts may be introduced early, as an underlying principal of metabolism [84, 85], or applied specifically to the electron transport chain (ETC) of mitochondria and chloroplasts [86]. It is here that students encounter the transduction of redox potential energy and membrane potential, and must integrate the language of redox biochemistry and electrophysiology. This kind of deep conceptual learning happens best when students solve problems or engage in other kinds of active-learning activities [87–89], which may now be augmented by generative artificial intelligence [90]. In the teaching laboratory, students can carry out voltammetric experiments on quinones to place electrochemical theory in a meaningful biological context [91, 92]. They may also investigate the ETC using spectrophotometry [93, 94] or Clark electrode polarography [95, 96]. These experiments lend themselves well to inquiry-based exercises: students can choose different redox-active dyes or enzyme inhibitors and measure their effects on particular parts of the ETC [94]. Finally, in advanced microbiological classes, electrogenic bacteria have been used to construct and operate a microbial fuel cell [97].

## Conclusions and recommendations

Bioelectrochemistry is a broad, interdisciplinary field that is poorly defined at the undergraduate level. It is widely taught, however, to students of both the chemical and the biological sciences, and appears in several parts of these curricula. The preceding review of current practice and pedagogy suggests the following recommendations for educators involved in bioelectrochemical instruction:

1. **Promote curricular integration.** Educators should familiarize themselves with the broader bioelectrochemical curriculum at their institution. Students learn how to apply electrochemical ideas to biology in different classes and in different ways, and teachers who make explicit references to these connections can help students develop an integrated understanding of the field. (For those working in the UK, bioelectrochemistry offers a good opportunity to address the “bringing together information and ideas from different topics” question in the National Student Survey [98].)
2. **Clarify terminology.** Highlight the differences in electrochemical language and notation when the same concept appears in different contexts. Care should be taken with the Nernst equation, which is used differently by chemists and biologists, to pre-empt confusion and support conceptual clarity.
3. **Use vivid**,** biologically relevant examples.** Practical illustrations of the connections between electrochemistry and biology can fire students’ enthusiasm for material that often seems abstract. Examples range from neural implants to artificial leaves, and demonstrations can help too – but beware the lemon cell, which may reinforce misconceptions.
4. **Use active learning strategies.** Inclusive teaching techniques with demonstrated utility in science education [99–101] should be used to make some interdisciplinary connections deep and detailed. For example, a mitochondrial case study in a general chemistry class could help students apply electrochemical principles and methods to a biological context.
5. **Incorporate practical exercises.** Educators should adapt published teaching laboratory activities that allow students to apply bioelectrochemical theory. Static electrochemical techniques and conductivity measurements are options for large classes, but low-cost hardware and open-source software is expanding access to bioelectrochemical analysis. Advanced students may benefit from more sophisticated equipment and techniques, including computer simulations when appropriate.

Bioelectrochemistry offers rich educational opportunities across the chemical and biological sciences. By addressing its conceptual, terminological, and curricular challenges, educators can help students build a coherent and engaging understanding of the field. Strengthening undergraduate bioelectrochemical education will enhance scientific literacy and support the development of future researchers and professionals.

## Data Availability

The informal survey data summarised in this article are not publicly available to preserve respondent confidentiality but are available in anonymized form from the author on reasonable request.

## Abbreviations

ECG: Electrocardiogram
ETC: Electron transport chain
PSRB: Professional, statutory and regulatory body
QAA: Quality Assurance Agency for Higher Education
RSB: Royal Society of Biology
UK: United Kingdom of Great Britain and Northern Ireland
USA: United States of America
